# *Bifidobacterium animalis* ssp. *lactis* CNCM-I2494 Restores Gut Barrier Permeability in Chronically Low-Grade Inflamed Mice

**DOI:** 10.3389/fmicb.2016.00608

**Published:** 2016-05-06

**Authors:** Rebeca Martín, Laure Laval, Florian Chain, Sylvie Miquel, Jane Natividad, Claire Cherbuy, Harry Sokol, Elena F. Verdu, Johan van Hylckama Vlieg, Luis G. Bermudez-Humaran, Tamara Smokvina, Philippe Langella

**Affiliations:** ^1^Commensal and Probiotics-Host Interactions Laboratory, Micalis Institute, Institut National de la Recherche Agronomique, AgroParisTech, Université Paris-SaclayJouy-en-Josas, France; ^2^Danone Nutricia ResearchPalaiseau, France; ^3^Farncombe Family Digestive Health Research Institute, McMaster UniversityHamilton, ON, Canada; ^4^ERL INSERM U 1057/UMR7203, Faculté de Médecine Saint-Antoine, Université Pierre et Marie CurieParis, France; ^5^Service de Gastroentérologie, Hôpital Saint-Antoine, Assistance Publique – Hôpitaux de ParisParis, France

**Keywords:** micro-inflammation, apical junction proteins, goblet cells

## Abstract

Growing evidence supports the efficacy of many probiotic strains in the management of gastrointestinal disorders associated with deregulated intestinal barrier function and/or structure. In particular, bifidobacteria have been studied for their efficacy to both prevent and treat a broad spectrum of animal and/or human gut disorders. The aim of the current work was thus to evaluate effects on intestinal barrier function of *Bifidobacterium animalis* ssp. *lactis* CNCM-I2494, a strain used in fermented dairy products. A chronic dinitrobenzene sulfonic acid (DNBS)-induced low-grade inflammation model causing gut dysfunction in mice was used in order to study markers of inflammation, intestinal permeability, and immune function in the presence of the bacterial strain. In this chronic low-grade inflammation mice model several parameters pointed out the absence of an over active inflammation process. However, gut permeability, lymphocyte populations, and colonic cytokines were found to be altered. *B. animalis* ssp. *lactis* CNCM-I2494 was able to protect barrier functions by restoring intestinal permeability, colonic goblet cell populations, and cytokine levels. Furthermore, tight junction (TJ) proteins levels were also measured by qRT-PCR showing the ability of this strain to specifically normalize the level of several TJ proteins, in particular for claudin-4. Finally, *B. lactis* strain counterbalanced CD4^+^ lymphocyte alterations in both spleen and mesenteric lymphoid nodes. It restores the Th1/Th2 ratio altered by the DNBS challenge (which locally augments CD4^+^ Th1 cells) by increasing the Th2 response as measured by the increase in the production of major representative Th2 cytokines (IL-4, IL-5, and IL-10). Altogether, these data suggest that *B. animalis* ssp. *lactis* CNCM-I2494 may efficiently prevent disorders associated with increased barrier permeability.

## Introduction

The intestinal barrier is an effective defense mechanism that depends on the integrity of the cells and the junctional complexes between them. The gut barrier is a functional unit organized as a multilayer system composed by a physical barrier which prevents bacterial adhesion and regulates paracellular diffusion and a functional layer able to discriminate between pathogens and commensal microorganisms (Lopetuso et al., 2015). The physical barrier is formed by a mucus layer followed by a monolayer of epithelial cells (Denker and Nigam, 1998; Natividad and Verdu, 2013) performing the paracellular transport across the barrier controlled by apical junction proteins (Natividad and Verdu, 2013). The mucus protects the epithelium from harmful microorganisms and antigens being also a lubricant for intestinal motility (Lopetuso et al., 2015). Outer mucus is composed of the highly glycosylated mucin MUC2 protein produced by the goblet cells (Lopetuso et al., 2015). The regulation of its function is mediated by both endogenous and exogenous factors (Agostini et al., 2012; Distrutti et al., 2013) and is a key factor in the development of several diseases involving altered gut permeability and dysfunction such as irritable bowel syndrome (IBS), food allergies, type-1 diabetes, and obesity (Perrier and Corthésy, 2011; Camilleri et al., 2012; Vaarala, 2012). Diverse microorganisms have shown to protect barrier integrity and promote its restoration when damaged. Among them, increasing evidence points out that strains of lactic acid bacteria (Gaudier et al., 2004) and bifidobacteria regulate gut barrier function using different mechanisms (Agostini et al., 2012; Distrutti et al., 2013). For instance, *Lactobacillus rhamnosus* GG (LGG), *B. breve* NCC2950 and a mixture of lactobacilli and bifidobacteria (*L. casei, L. plantarum, L. acidophilus, L. delbrueckii ssp. bulgaricus, B. longum*, *B. breve, and B. infantis*) prevent the increase in intestinal permeability *in vivo* (Ukena et al., 2007; Mennigen et al., 2009; Donato et al., 2010; Natividad et al., 2013).

Bifidobacteria, naturally present in the colonic microbiota, correspond to up to 80% of the cultivable fecal microorganisms in full-term breastfed infants (Picard et al., 2005). They have been traditionally considered as safe microorganisms, due to their Generally Recognized As Safe (GRAS) status and are widely used as health-promoting bacteria in functional foods. Especially, *B. animalis* ssp. *lactis* (*B. lactis*) CNCM I-2494 has a long history of use in fermented dairy products and shows a high gastrointestinal survival (Picard et al., 2005; Rochet et al., 2008). A fermented milk product (FMP)-containing *B. lactis* CNCM I-2494 together with lactic acid bacterial starter cultures has shown positive effects on gut function in several randomized controlled studies (Picard et al., 2005) improving: (i) gastrointestinal well-being and digestive symptoms in women reporting minor digestive problems (Guyonnet et al., 2009a), (ii) abdominal girth and gastrointestinal transit (Agrawal et al., 2009), (iii) health related quality of life and symptoms in IBS in adults (Guyonnet et al., 2009b), and (iv) colonic transit time and minor digestive problems in healthy women (Marteau et al., 2002, 2013). The physiological effects of this strain have been also evaluated in animal studies where it has been capable to reduce the aberrant crypts incidence in chemically induced carcinogenesis models in rats (Tavan et al., 2002), improve colitis in mice (Veiga et al., 2010), hydrolyze bile salts in the gastrointestinal tract of pigs (Lepercq et al., 2004), and prevent the increase of intestinal permeability induced by partial restraint stress in rats (Agostini et al., 2012). The molecular mechanisms underlying the positive effects of strain CNCM I-2494 are far from being completely understood although its genome have been sequenced (Chervaux et al., 2011). Recent identification of several restriction and modification systems in this strain and development of specific molecular tools opened the way in studying specific bacterial mechanisms involved in the cross-talk of strain CNCM I-2494 with the host (O’Connell Motherway et al., 2014).

The clear relationship between *B. lactis* CNCM I-2494 and the protection of gut dysfunction in both animal models and clinical trials combined to the industrial importance of this strain has prompted us to deeper analyze its possible effects on an altered permeability and gut dysfunction model. Gut dysfunction was achieved thanks to a first inflammatory insult followed with a second subclinical chemical challenge as previously described (Laval et al., 2015; Martin et al., 2015). The aim of this work was to clarify the direct effect of the strain in the murine intestinal epithelium barrier and function.

## Materials and Methods

### Bacterial Growth Conditions and Animals

*Bifidobacterium animalis* ssp. *lactis* CNCM-I2494 was grown in MRS medium (Difco, USA) supplemented with cysteine (0.5 mg/ml; Sigma–Aldrich) under anaerobic conditions at 37°C.

Male C57BL/six mice (6–8 weeks old; Janvier, Le Genest Saint Isle, France) were maintained at the animal care facilities of the National Institute of Agricultural Research (IERP, INRA, Jouy-en-Josas, France) under specific pathogen-free (SPF) conditions. Mice were housed under standard conditions for a minimum of 1 week before experimentation. All experiments were performed in accordance with European Community rules for animal care and were approved by the relevant local committee (Comethea). Protocol number 02550.01.

### Experimental Design

Inflammation was induced as previously described (Laval et al., 2015) (**Supplementary Figure S1**). Briefly, mice where challenged, under anesthesia, with a first intra-rectal dose of 100 mg/Kg of dinitrobenzene sulfonic acid (DNBS) solution (ICN, Biomedical Inc.) in 30% ethanol (EtOH). Control mice (without colitis) received only 30% EtOH. Thirteen days after the first DNBS injection, 5 × 10^9^ CFU of viable bacteria in 200 μl of PBS or PBS alone were administered intra-gastrically, daily for 10 days (gavage period). Finally, 21 days after the first challenge, the mice were challenged again with a second administration of 50 mg/kg of DNBS or EtOH. Weight loss was monitored during 3 days following the second DNBS injection to assess possible clinical signs of distress.

To confirm the absence of over inflammation, colonic macro scopic and histological scores as well as colonic myeloperoxidase (MPO) activity (a marker of the degree of infiltration by polymorphonuclear neutrophils) and serum lipocalin-2 levels (an early inflammation marker) were determined as previously described (Shashidharamurthy et al., 2013; Martin et al., 2014; Laval et al., 2015).

### Histological Features Analysis

Flushed colons were fixed in 4% paraformaldehyde or Carnoy buffer, dehydrated and embedded in paraffin according to a standard protocol. Histological features were analyzed by hematoxylin–eosin–safran (Perrier and Corthésy, 2011) staining. Periodic acid-Schiff (PAS) and Alcian blue (AB) staining were performed as in Wrzosek et al. (2013).

### Intestinal Permeability *In Vivo*

Permeability *in vivo* was assessed using fluorescein isothiocyanate-conjugated dextran (FITC–dextran 3000–5000 Da, Sigma–Aldrich) tracer as previously described (Tambuwala et al., 2010). Briefly, at the endpoint 0.6 mg/g body weight of FITC–dextran dissolved in PBS was administered to mice by oral gavage. To measure the presence of FITC–dextran in blood, 3.5 h after the gavage blood samples were recovered from the retro-orbital venous plexus and kept in dark at 4°C until analysis. Mice were housed under standard conditions during this period with un-limited access to water and food. Serum has separated by centrifugation and plasma FITC levels were determined using a fluorescence microplate reader (excitation 485 nm and emission 530 nm; Tecan, Lyon, France).

### Apical Junctional Analysis by Quantitative Real-time PCR (qPCR)

Total RNA was isolated from 20 to 30 mg samples of colon with an RNeasy Mini Kit (Qiagen) as previously described (Laval et al., 2015). qPCR was performed with diluted cDNA (10×) in triplicate and with an iQ5 Real-Time Detection System (Bio-Rad). The reaction mix consisted of Ssofast Evagreen Supermix (Bio-Rad), primers at 0.5 μM (Martin et al., 2015), and 2 μL of diluted cDNA. Values are expressed as relative fold differences normalized to a housekeeping gene, *Gapdh*, by the 2^−ΔΔC_T_^ method. All procedures were performed according to the manufacturers’ instructions.

### Analyses of Lymphoid Populations Present in the Spleen and in the Mesenteric Lymphoid Nodes (MLNs)

Mononuclear cells were isolated from spleens and MLN by gentle extrusion of the tissue through a 50 μm-mesh Nylon cell strainer (BD). Cells were suspended in Dulbecco’s Modified Eagle Medium (DMEM) medium supplemented with 10% of fetal calf serum (FCS), 2 mM L-glutamine, 50 U/mg penicillin, and 50 U/mg streptomycin (Lonza, Levallois-Perret, France). Erythrocytes were lysed with red blood-cell lysing buffer (Sigma–Aldrich).

For flow cytometry analysis, aliquots of 10^6^–10^7^ cells per sample were pre-incubated with purified anti-mouse CD16/CD32 (eBioscience, San Diego, CA, USA) and then labeled with anti-CD4-FITC, anti-CD3e-PE, and anti-CD8-PerCP (all from eBioscience) according to the manufacturer’s instructions. The stained cells were analyzed by flow cytometry (Accuri, BDbioscience) with CFlow Sampler software (BD).

For stimulation experiments, 2 × 10^5^ cells per well were cultured for 48 h (37°C, 10% CO_2_) in DMEM medium in P24 plates pre-coated with anti-CD3/CD28 antibodies (4 μg/mL each; eBioscience) or phorbol 12-myristate 13-acetate (PMA)/ionomycin (cell stimulation cocktail, 1×, ebioscience). Culture supernatant was frozen at −80°C until processing.

### Cytokine Assays

Blood samples were obtained from the retro-orbital venous plexus before the mice were euthanized and centrifuged, and the sera stored at −80°C until analysis. One centimeter samples of distal colon were recovered and homogenized in an appropriate volume of PBS (final concentration of 50 mg/ml) in a Tissue Lyser (Qiagen). IL-6, IL-10, IFN-γ, TNF-α, IL-5, IL-2, IL-22, IL-1α, IL-13, IL-17, IL-4, IL-27, and IL-12p70 were assayed in blood and colon samples with a cytometric bead array system (Mouse Th1/Th2/Th17/Th22 13plex Flowcytomix; eBioscience, San Diego, CA, USA). For cytokine quantification in cell culture supernatants the following ELISA tests were performed according to manufacturer’s instruction: IL-4, IL-5, IFNγ, IL-17, IL-12p70, and IL-10 (MabTech); TGFβ and IL-22 (ebioscience).

### Statistical Analysis

GraphPad software (GraphPad Sofware, La Jolla, CA, USA) was used for statistical analysis. Results are presented as bar graphs or dot plots with means ± SEM. Comparisons involved the non-parametric Kruskal-Wallis test followed by a Dunn’s Multiple Comparison test. A *p* value of less than 0.05 was considered significant.

## Results

### Confirmation of Micro-inflammation in DNBS Challenged Mice

The induction of a low-grade inflammation status following a chronic low-dose DNBS in the mice was confirmed through the follow-up of health, histological and inflammatory parameters (**Supplementary Figure S1**). In particular, weight loss (**Supplementary Figure S2A**), colonic macroscopic and histological scores (**Supplementary Figures S2B,C**) as well as the MPO activity in the colon (**Supplementary Figure S2D**) and the Lipocalin-2 concentration in serum (**Supplementary Figure S2E**) were measured. The absence of differences for all these parameters among the groups, even in presence of *B. lactis* CNCM-I2494, added to the lack of detection of cytokine levels in serum samples (IL-6, IL-10, IFN-γ, TNF-α, IL-5, IL-2, IL-22, IL-1α, IL-13, IL-17, IL-4, IL-27, and IL-12p70, data not shown) discards the presence of an overt and active inflammation in this model.

**FIGURE 1:**
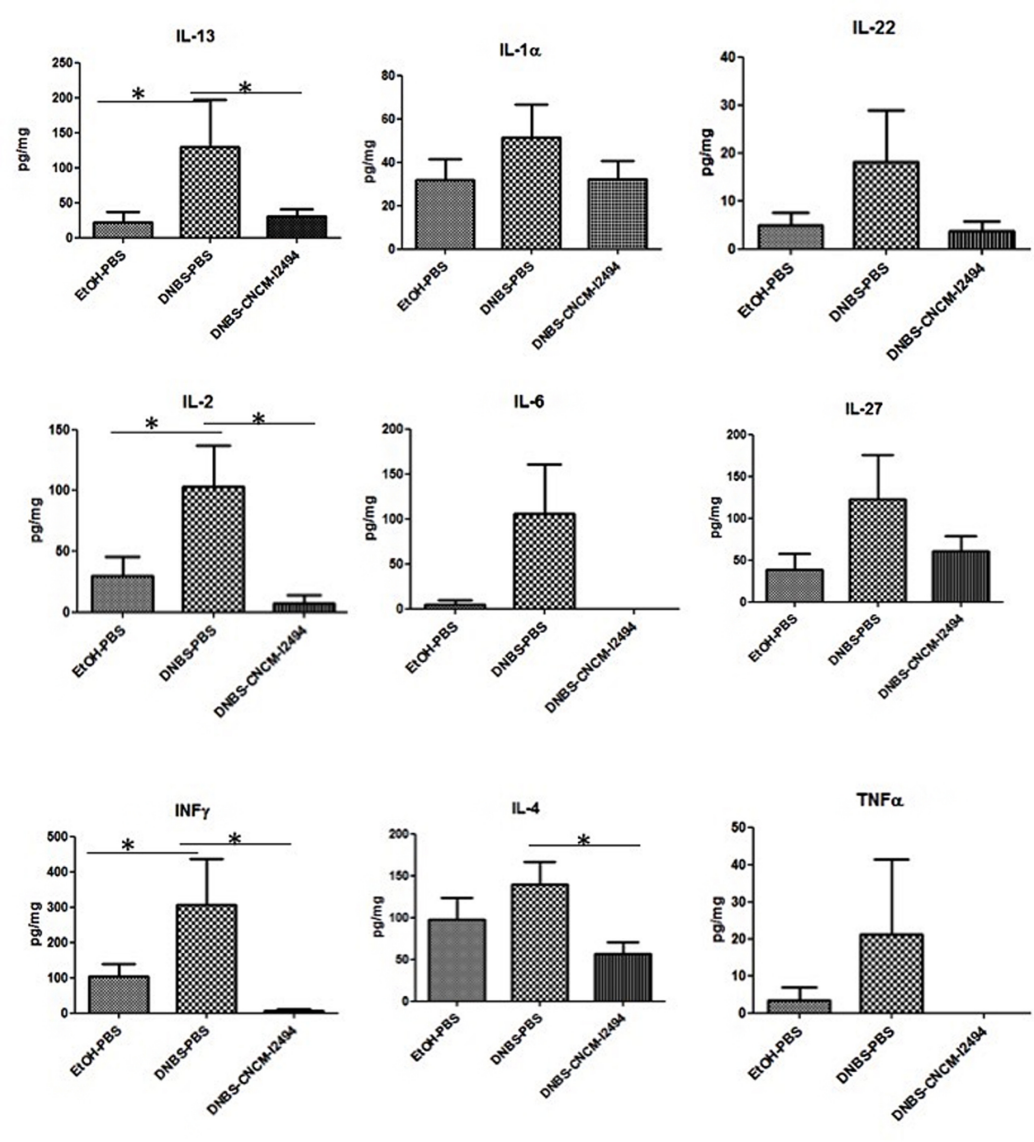
**Cytokine concentrations in colon in the dinitrobenzene sulfonic acid (DNBS) micro-inflammation model**. Control non-inflamed (EtOH–PBS), control inflamed (DNBS–PBS), and *B. lactis* CNCM I-2494 strain (DNBS–CNCM-I2494). ^∗^*p <* 0.05 (*n* = 8).

However, the presence of slightly elevated, although no statistically significant, cytokines IL-13, IL-1α, IL-6, IL-22, IL-2, IL-27, IL-4, IFN-γ, and TNF-α levels in colonic tissues, compared to healthy controls, suggest a local low-grade inflammation (**Figure 1**). Treatment with *B. lactis* CNCM-I2494 reduced these increases in cytokine production (**Figure 1**): Notably, restoration was statistically significant for IL-2, IL-13, and IFNγ (*p* < 0.05).

### *Bifidobacterium lactis* CNCM-I2494 Restores Colonic Permeability by Modulating Apical Junction Protein Levels

**FIGURE 2:**
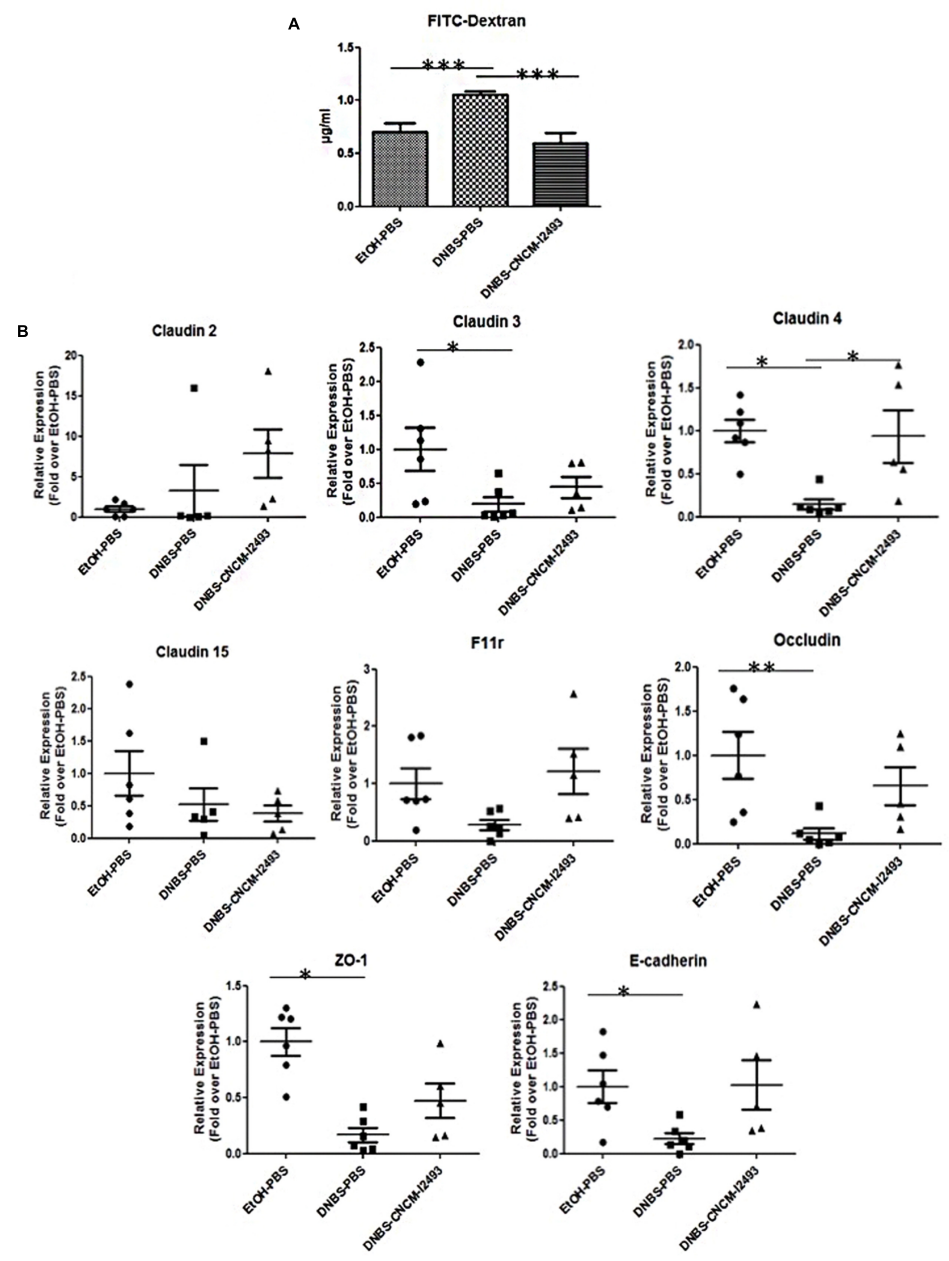
***In vivo* permeability measurements and effect on apical junction protein mRNAs**. For *in vivo* measurements of gut permeability, animals were orally gavaged with fluorescein isothiocyanate (FITC)-dextran **(A)**. Apical junction protein expression levels were determined by real-time qPCR **(B)**. Control non-inflamed (EtOH-PBS, black circles) control inflamed (DNBS-PBS, black squares) *B. lactis* CNCM I-2494 strain (DNBS-CNCM-I2494, black triangles). ^∗^*p <* 0.05, ^∗∗^*p <* 0.01, ^∗∗∗^*p <* 0.001 (*n* = 8).

The integrity of the gut barrier was assessed by the analysis of the permeability with the paracellular tracer FITC-dextran *in vivo* at the endpoint. Of note, all the animals were submitted to exactly the same protocol and waiting time to avoid differences due to a minimal possible clearance phenomenon due to renal function. The mice treated with DNBS showed high permeability to the tracer (*p* < 0.05) (**Figure 2A**) confirming an alteration in the barrier permeability as it has been previously observed (Laval et al., 2015; Martin et al., 2015). The oral administration of *B. lactis* CNCM-I2494 strain resulted in a decrease in permeability (*p* < 0.05). To further analyze the effect on the barrier function the expression of the relevant mRNAs of adherent junction (AJ) and tight junction (TJ) proteins were measured by qRT-PCR (**Figure 2B**). The mRNAs for Claudin-3, 4, E-cadherin, Occludin, and the zona occludens proteins (ZO-1) were all less abundant in DNBS-treated mice than in control mice (*p* < 0.05). CNCM-I2494 tends to partially re-establish the levels of all of them (**Figure 2B**). Notably, this effect was statistically significant for Claudin-4 (*p* < 0.05). Taken together, both the histological analysis and the transcriptional data demonstrate that strain *B. lactis* CNCM I-2494 protects against DNBS-induced chronic barrier dysfunction.

### *Bifidobacterium lactis* CNCM-I2494 Restores Goblet Cell Population Altered by DNBS Chronic Challenge

**FIGURE 3:**
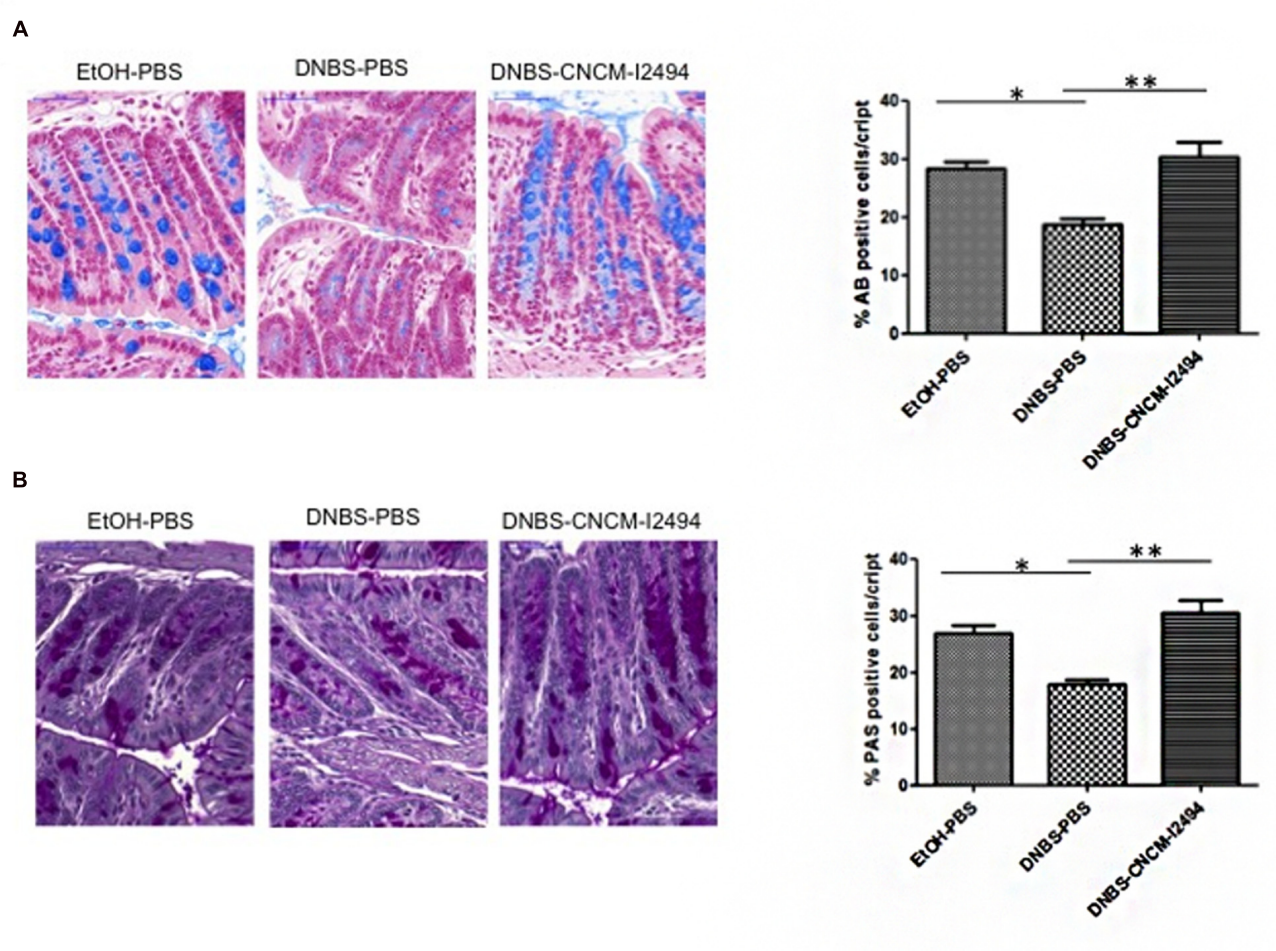
**Goblet cell detection**. Representative photos and % of positive cells stained with AB (Alcian Blue) **(A)** and PAS (Periodic Acid-Schiff) **(B)**. Control non-inflamed (EtOH–PBS), control inflamed (DNBS–PBS), *B. lactis* CNCM I-2494 strain (DNBS–CNCM-I2494). ^∗^*p <* 0.05 ^∗∗^*p <* 0.01 (*n* = 4).

Histological features, analyzed by hematoxylin–eosin–safran (Perrier and Corthésy, 2011) staining, showed no significant differences in general morphology, crypt depth or total numbers of cells per crypt (data not shown). The numbers of goblet cells stained either by AB (**Figure 3A**), specific for acidic mucopolysaccharides, or PAS (**Figure 3B**), specific for neutral mucopolysaccharides, were significantly lower in DNBS challenged control group (*p* < 0.05). *B. lactis* CNCM-I2494 was able to enhance the percentage of AB or PAS positive cells per crypt (*p* < 0.05) reaching the values of the non-inflamed control group (**Figure 3**).

### *Bifidobacterium. lactis* CNCM I-2494 Modulates CD3^+^/CD4^+^ T-Cell Populations in Spleen and MLNs by Increasing T Helper (Th) Profile 2

**FIGURE 4:**
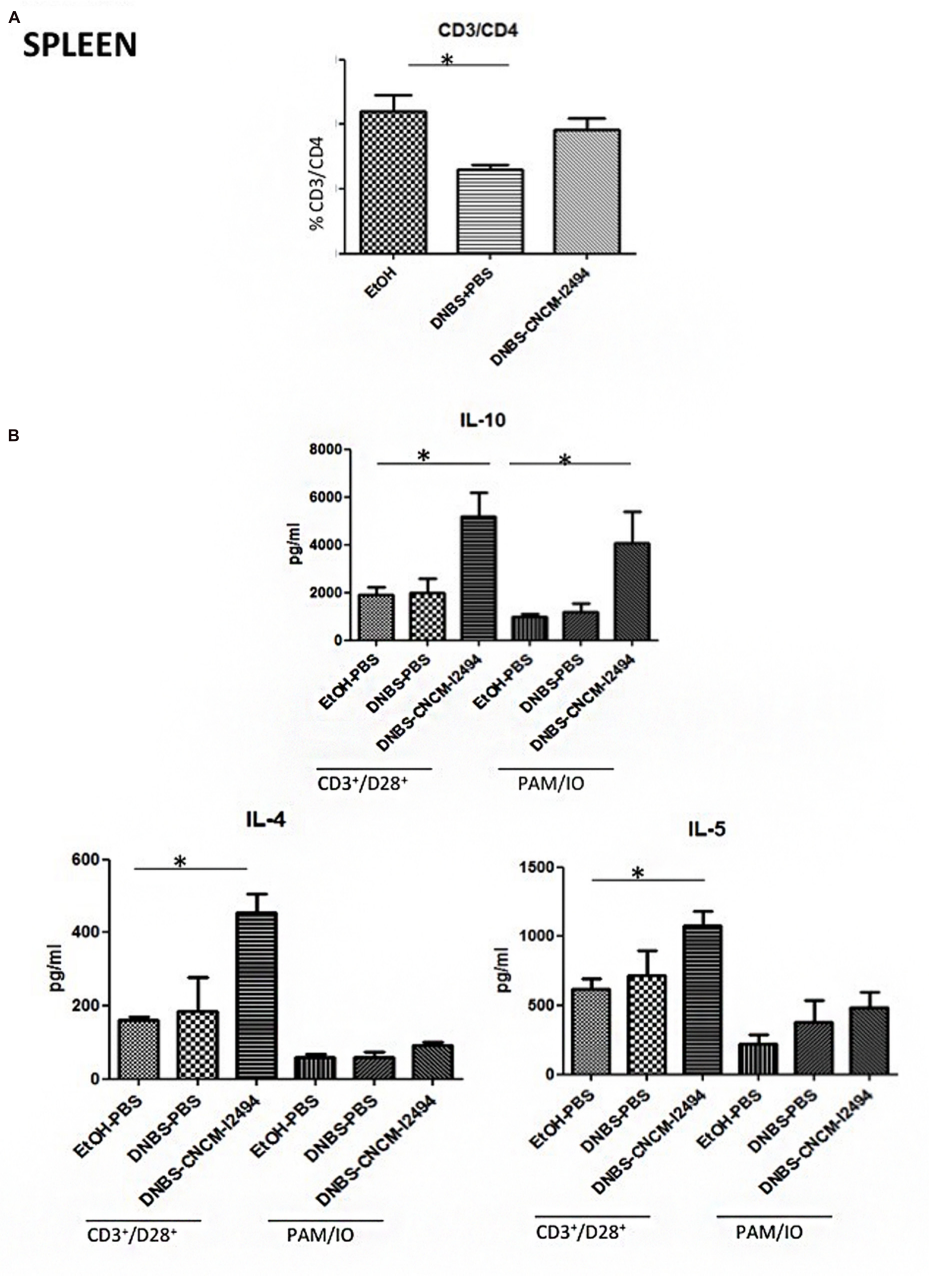
**Splenocyte population levels**. CD3/CD4 positive cells detected by flow cytometry **(A)** and cytokine production in spleen cell cultures stimulated with CD3^+^/CD28^+^ or PAM/IO **(B)**. Control non-inflamed (EtOH-PBS), control inflamed (DNBS-PBS), *B. lactis* CNCM I-2494 strain (DNBS-CNCM-I2494). ^∗^*p <* 0.05 (*n* = 8).

**FIGURE 5:**
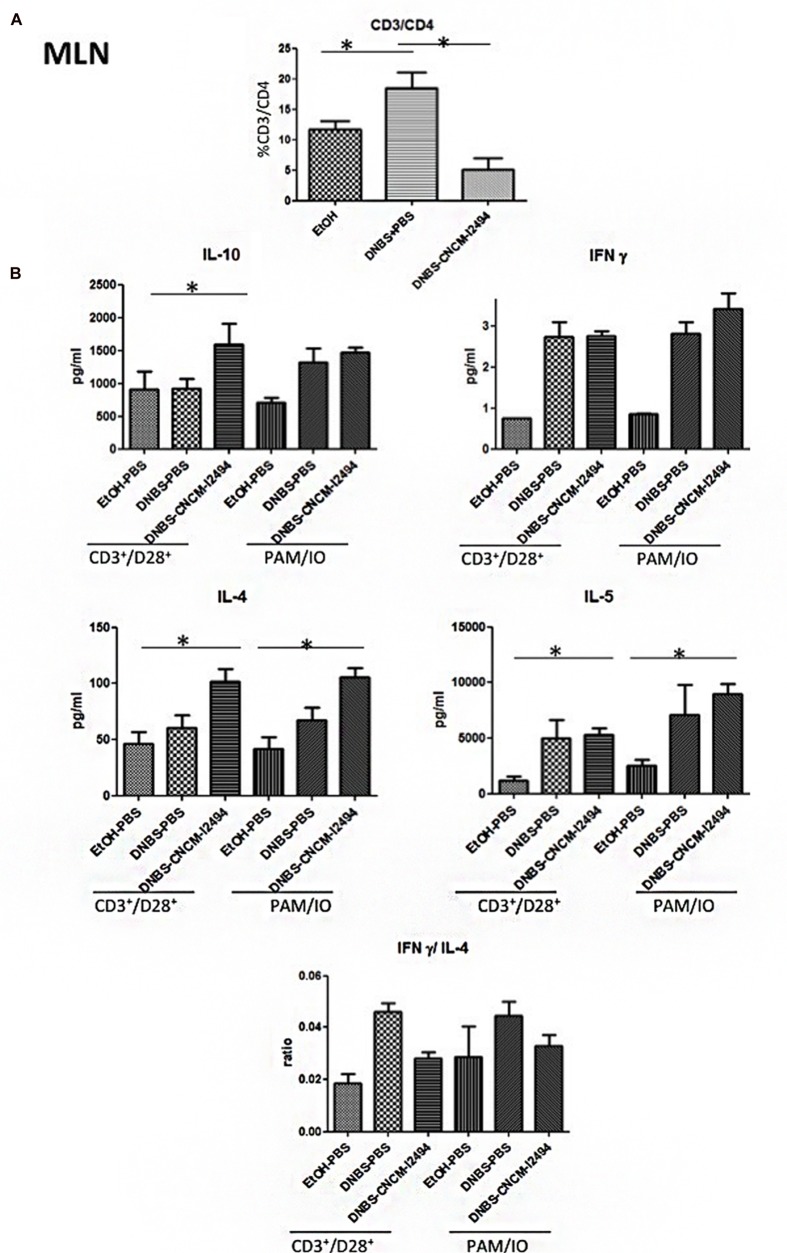
**MLN population levels**. CD3/CD4 positive cells detected by flow cytometry **(A)** and cytokine production in MLN cultures stimulated with CD3^+^/CD28^+^ or PMA/IO **(B)**. Control non-inflamed (EtOH–PBS), control inflamed (DNBS–PBS), *B. lactis* CNCM I-2494 strain (DNBS–CNCM-I2494). ^∗^*p <* 0.05 (*n* = 8).

To study further the mechanism by which *B. lactis* CNCM-I2494 exerts protective function, T-cells from spleen and MLN were isolated and analyzed by flow cytometry. DNBS-treated mice showed lower CD3^+^/CD4^+^ T-cell percentages in spleen (**Figure 4A**) than the control group and higher CD3^+^/CD4^+^ cell percentages in MLN (**Figure 5A**; *p* < 0.05). *B. lactis* CNCM-I-2494 tends to reduce the CD3^+^/CD4^+^ decrease in spleen (**Figure 4A**) and significantly control CD3^+^/CD4^+^ increase in MLN (*p* < 0.05; **Figure 5A**). No variations were observed in CD3^+^/CD8^+^ T-cell percentages in spleen or MLN (data not shown).

As variations in CD4^+^ T-cell populations were found, MLN and spleen cells were cultured after isolation in the presence of two different stimulators during 48 h: CD28^+^/CD3^+^ to specifically stimulate lymphocytes and PMA/IO to stimulate all the cells present in the organ disaggregate. Representative cytokines of the major Th profiles (IL-4, IL-5, IFNγ, IL-17, IL-12p70, IL-10, TGFβ, and IL-22) were determined in the culture supernatants (**Figures 4B** and **5B** and data not shown). The IL-17, IL-22, IL-12p70, and TGFβ levels as well as IFNγ in spleen samples were under the ELISA detection limits (2.4, 5.5, 8.6, 10, and 6.5 pg/ml, respectively; data not shown). This fact, in addition to slight increases of Th1 levels (IFNγ) by the DNBS treatment in MLN (**Figure 5B**) confirms the low-grade inflammation status of the mice model. Differences were found in the levels of IL-4, IL-5, and IL-10 in both spleen (**Figure 4B**) and MLN (**Figure 5B**) (*p* < 0.05). Strain CNCM-I2494 increased Th2 levels as measured by IL-4 and IL-10 augmentation in both spleen (**Figure 4B**) and MLN (**Figure 5B**) and also IL-5 in spleen samples corresponding to an anti-inflammatory patter in this model. This anti-inflammatory patter has been confirmed locally by the INFγ/IL-4 ratio in MLN samples (**Figure 5B**). Nevertheless, CNCM-I2494 was not able to control the small increase in IFNγ caused by the DNBS challenge (**Figure 5**). Finally, DNBS treatment caused also an increase in IL-5 in MLN samples. No significant differences were found between CD3^+^/CD28^+^ and PMA/IO stimulations, excepting IL-4 and IL-5 in spleen where a major level of stimulation was achieved with the first one (**Figures 4** and **5**). Taken together these data demonstrate that CNCM-I2494 strain is able to counterbalance the Th1/Th2 ratio altered by the DNBS challenge (which locally augments CD4^+^ Th1 cells) by increasing the Th2 response as measured by the increase in the production of major representative Th2 cytokines.

## Discusion

Epithelial barrier dysfunction is now considered as one of the major contributors to the development of several diseases and syndromes (Perrier and Corthésy, 2011; Camilleri et al., 2012; Vaarala, 2012). In several of them, such as IBS, studies suggest an interplay between luminal factors (e.g, foods and bacteria residing in the intestine), the epithelial barrier, and the mucosal immune system (Barbara et al., 2012). In a healthy state, the epithelial barrier allows a low translocation of luminal antigens by paracellular transport by receptor-mediated or non-selective endocytosis (Natividad and Verdu, 2013). Therefore, a higher local antigen exposure caused by an increase of intestinal permeability could activate intestinal immune system and inflammation may thus occur (Ohman and Simren, 2007; Natividad and Verdu, 2013). Preclinical studies have shown that selective probiotic strains exhibit the potential to improving mucosal barrier homeostasis (Barbara et al., 2012).

As related above, the administration of fermented milk containing *B. lactis* CNCM I-2494 has been found to prevent *in vivo* the increase of intestinal permeability in rats (Agostini et al., 2012). However, due to possible synergistic interplay of the different strains and/or metabolites contained in this product the specific effect of this *B. lactis* strain on gut barrier is still unknown. Here, we aimed to clarify the specific effect of *B. lactis* CNCM-I2494 strain on intestinal barrier function.

As previously observed, DNBS-treated mice showed alteration in gut permeability (Laval et al., 2015). *In vivo* values with the paracellular tracer FITC-dextran showed an increase in permeability in DNBS-challenged mice that was restored when mice were treated with *B. lactis* CNCM-I2494 suggesting an effect of the strain on gut barrier function. To better decipher the beneficial effect of *B. lactis* CNCM-I9434 strain on gut permeability, mucus producing cells were analyzed by two different specific staining: AB, which specifically stains acidic mucopolysaccharides and PAS staining, specific for neutral mucopolysaccharides. Both staining protocols reveal the decrease of goblet cell mucus producing cells in mice challenged with DNBS, confirming the functional abnormalities on the tissue despite the lack of macroscopic or microscopic damages. Mice treated with *B. lactis* CNCM-I2494 strain recover the same goblet cell accounts than control mice pointing out a positive effect of the strain in restoring epithelial normal cell composition and probably the mucus production. In fact, mucus production was previously shown to be affected during inflammation with intestinal dysbiosis (Fyderek et al., 2009). Several bifidobacteria strains, alone or in mixture, induce mucus production and/or are able to adhere to it (He et al., 2001; Gaudier et al., 2005). Even if the goblet cell depletion observed in DNBS challenged mice could explain the differences found in *in vivo* permeability, alterations in apical junction proteins have been also reported previously in this model (Laval et al., 2015). The apical junctions are formed by TJ and AJ proteins. Here, according to our previous results, the expression of TJ proteins measured by RT-qPCR is reduced by the DNBS intra-rectal administration in a protein-specific way (Laval et al., 2015). The treatment with *B. lactis* CNCM-I2494 strain tended to restore F11r, Occludin, E-cadherin and ZO-1 expression, showing this effect especially remarkable for claudin 4. These results are consistent with previous studies in which some lactic bacteria and bifidobacteria prevented changes in occludin, ZO-1, claudin-1, claudin-3, claudin-4, and claudin-5 proteins (Mennigen et al., 2009). Indeed, Agostini et al. (2012) showed that *B. lactis* CNCM-I2494 restored occludin and JAM-A concentrations to control levels after partial restrain stress in rat administration of fermented milk containing *Lactococcus lactis* CNCM-I1631 and two classical yogurt starters.

Changes on mucosal permeability as the ones observed in the DNBS low-dose model can be the cause or the consequence of a low immune activation. To assess the effect of *B. lactis* CNCM-I2494 strain on mucosal immunity and decipher its possible effect on host immune response, colonic cytokine levels, and spleen MLN lymphocyte populations were analyzed. In this specific context, *B. lactis* CNCN-I2494 treatment restored the mild increased IL-13, IL-2, IL-4, and INF-γ colonic values to normal. Several studies pointed out the cytokines as one of the causes of TJ protein modulation. For instance, *in vitro* test have shown a relationship between IL-13 and an increase in paracellular permeability (Prasad et al., 2005) and INF-γ or IL-4 increases have been linked to TJ protein expression alterations (Bruewer et al., 2005; Wisner et al., 2008; Suzuki et al., 2011). Therefore, the effect of *B. Lactis* CNCM-I2494 on cytokine down-regulation could be the factor which triggered permeability restoration.

Mucosal dendritic cells present antigens to the adaptative immune system which directs the polarization of naïve CD4 T cells toward different T-helper cell subsets (Th1 and Th2 among others; Zhu and Paul, 2008). Classically, hapten-mediated colon inflammation protocols trinitrobenzene sulfonic acid (TNBS and DNBS) have been associated with Th1 response (Zuo et al., 2014). Our study confirms that, even in a gut dysfunction model provoked by a low-grade inflammation, DNBS challenge increase lightly Th1 response. Although *B. lactis* CNCM-I2494 was not able to decrease Th1 response, an upper-regulation of Th2 subset has been observed counterbalancing the Th1/Th2 ratio at local level. The increased Th2 cell subset may contribute to the decreased Th1 cell subset due to the mutual antagonizing effects of both Th substets (Donato et al., 2010). Several studies have been performed to assert the role of *Bifidobacterium* strains in modulating T-cell populations, being their results strain and model dependent (Lopez et al., 2011). Our results are consistent with those of Zheng et al. (2014) who showed that one strain of *B. breve* modulates T cell polarization toward Th2 and Treg cell-associated responses *in vitro* and *in vivo i*n a murine model of DSS-induced colitis.

Our results support the hypothesis of Agostini et al. (2012) who pointed out the improvement of the intestinal barrier (epithelial cells and mucus layers) permeability as part of the beneficial effect of the fermented milk commercial product containing CNCM-I2494. In addition, here we firstly point to CNCM-I2494 strain as a possible responsible of this effect. Furthermore, the present study supports that the action mechanism of this protective effect may be mediated by improvement on apical junction proteins and goblet cell population. Finally, the modulation of the host T-cell composition by CNCM-I2494 strain may be the host pathway involved in this phenomenon.

## Author Contributions

RM, TS, JH, EV, CC, LB-H, and PL designed all the experiments. RM, SM, FC, LL, JN, and HS performed the experiments. RM wrote the manuscript. SM, TS, JH, and PL corrected the manuscript. All authors read and approved the final manuscript.

## Conflict of Interest Statement

The authors declare that the research was conducted in the absence of any commercial or financial relationships that could be construed as a potential conflict of interest.

## Supplementary Material

FIGURE S1 **Low-grade inflammation experimental protocol**. Colitis was induced by intra-rectal administration of 100 mg/kg of DNBS in solution in 30% ethanol. Control mice (without colitis) received only 30% EtOH. The effects of DNBS are highest during the first 3 days after its administration (DNBS period). Ten days after the end of the DNBS period bacterial culture or PBS were intra-gastrically administered daily for 10 days (gavage period). Colitis was reactivated 21 days after the first DNBS injection with a second injection of 50 mg/kg of DNBS solution. Three days after reactivation mice were sacrificed. Modified from Martin et al. (2015).

FIGURE S2 **Evaluation of inflammatory status of DNBS-challenged mice**. Inflammatory status assessed from **(A)** the recovery after reactivation (%weight change); **(B)** macroscopic score; **(C)** histological score; **(D)** MPO activity *n* = 24 mice per group, and **(E)** Lipocalin-2 levels (*n* = 8 mice per group) in the control non-inflamed group (EtOH–PBS), control inflamed group (DNBS–PBS), *B. lactis* CNCM I-2494 strain (DNBS–CNCM-I2494).
