# Supplementary figures and images for: Bifidobacterium animalis ssp. lactis CNCM-I2494 Restores Gut Barrier Permeability in Chronically Low-Grade Inflamed Mice
Source: Front Microbiol. 2016 May 6;7:608. doi: 10.3389/fmicb.2016.00608 (PMC4858658; doi:10.3389/fmicb.2016.00608)

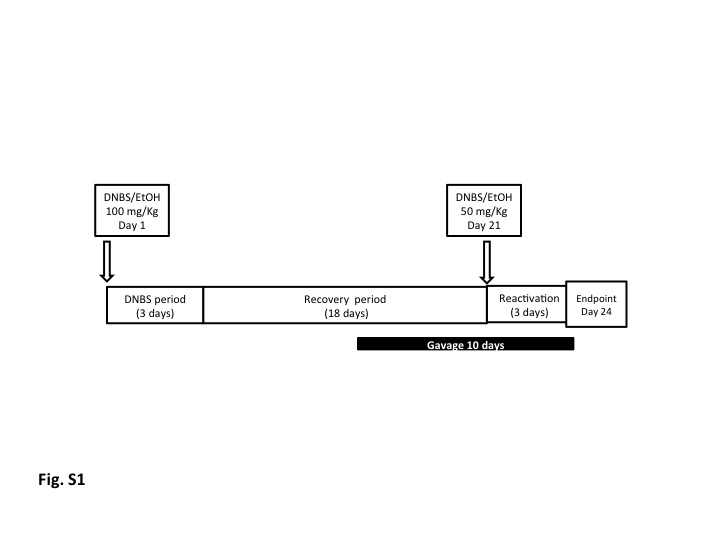

Supplement: FIGURE S1 — Low-grade inflammation experimental protocol. Colitis was induced by intra-rectal administration of 100 mg/kg of DNBS in solution in 30% ethanol. Control mice (without colitis) received only 30% EtOH. The effects of DNBS are highest during the first 3 days after its administration (DNBS period). Ten days after the end of the DNBS period bacterial culture or PBS were intra-gastrically administered daily for 10 days (gavage period). Colitis was reactivated 21 days after the first DNBS injection with a second injection of 50 mg/kg of DNBS solution. Three days after reactivation mice were sacrificed. Modified from Martin et al. (2015). [file Image_1.JPEG]

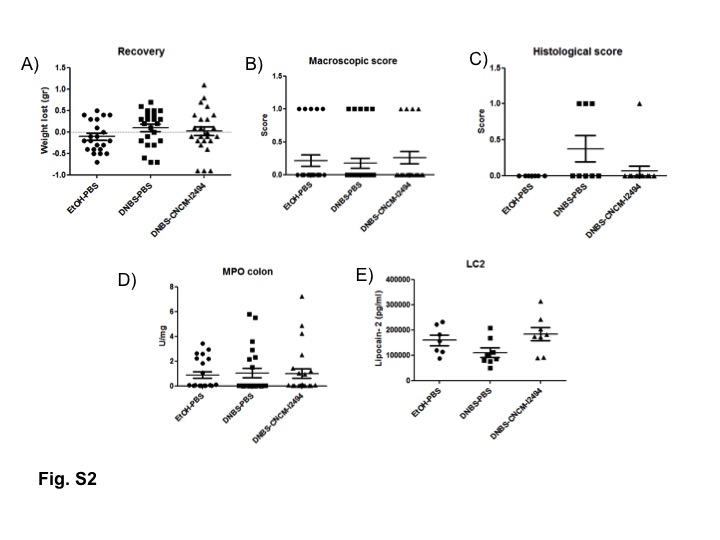

Supplement: FIGURE S2 — Evaluation of inflammatory status of DNBS-challenged mice. Inflammatory status assessed from (A) the recovery after reactivation (%weight change); (B) macroscopic score; (C) histological score; (D) MPO activity n = 24 mice per group, and (E) Lipocalin-2 levels (n = 8 mice per group) in the control non-inflamed group (EtOH–PBS), control inflamed group (DNBS–PBS), B. lactis CNCM I-2494 strain (DNBS–CNCM-I2494). [file Image_2.JPEG]
